# THE USE OF THE “TIME TRAVEL” PARADIGM OF ALZHEIMER’S DISEASE FOR FAMILY CARE PARTNER EMPOWERMENT

**DOI:** 10.1093/geroni/igad104.1514

**Published:** 2023-12-21

**Authors:** Christopher Johnson, Roxann Johnson, Fei Sun

**Affiliations:** Texas State University, San Marcos, Texas, United States; Aging Consultants Inc, Austin, Texas, United States; Michigan State University, East Lansing, Michigan, United States

## Abstract

This study describes how family carers are taught the Time Travel model of Alzheimer’s Disease (AD). As persons with AD experience cognitive, emotional, social, physical and functional time travel, families can learn to join them on their journey. Stage models (e.g., Reisberg and associates FAST and GDS) of AD clarify how changes through time occur using stage markers. Yet the disease is non-linear. Persons with AD don’t travel neatly in stages but fluctuate in recall of names, faces and events. They migrate in a non-linear downward spiral fluctuating back and forth through time, revisiting people, places, events and traumas of their distant past. Time Travel apps and other life history data are available to provide real person-centered care that’s time appropriate. Families are trained to connect in the approximate time frame in which the person has traveled. Hence, care partners are taught time appropriate communication and interventions (e.g., activities, pictures, music etc.) to respond to challenging behaviors. The Models use with family carers empowers them to validate their loved ones’ approximate time frame they are in. Family carers are inspired to become “best friends” of AD persons by understanding how this cognitive disability works. When a lady with travels to age 20 in her mind but sees an 80-year-old face in the mirror, she demands the person leave the bathroom. This behavior is puzzling to families. Time Travel model explains how this works driving care planning, redesigns (e.g., mirrors) and activities, and supports validation therapy, “Best Friends” and other pedagogy.

